# Effect of ABCB1 most frequent polymorphisms on the accumulation of bictegravir in recombinant HEK293 cell lines

**DOI:** 10.1038/s41598-024-66809-0

**Published:** 2024-07-15

**Authors:** Julien De Greef, Mathilde Akue, Nadtha Panin, Kévin-Alexandre Delongie, Marina André, Gwenaëlle Mahieu, Emilia Hoste, Laure Elens, Leïla Belkhir, Vincent Haufroid

**Affiliations:** 1 Louvain Centre for Toxicology and Applied Pharmacology, Institut de Recherche Expérimentale et Clinique, Université catholique de Louvain (UCLouvain), Brussels, Belgium; 2 Service de Médecine Interne et Maladies infectieuses, Cliniques universitaires Saint-Luc, Brussels, Belgium; 3 Department of Clinical Chemistry, Cliniques universitaires Saint-Luc, Brussels, Belgium; 4 Integrated PharmacoMetrics, PharmacoGenomics and PharmacoKinetics, Louvain Drug Research Institute, Université catholique de Louvain (UCLouvain), Brussels, Belgium; 5 Pharmacologie Cellulaire et moléculaire, Louvain Drug Research Institute, Université catholique de Louvain (UCLouvain), Brussels, Belgium

**Keywords:** Integrase strand transfer inhibitors, Pharmacogenetic, P-glycoprotein, P-gp, Multi-drug resistance protein 1, MDR1, HIV infections, Pharmacogenetics

## Abstract

Bictegravir, a key second-generation integrase strand transfer inhibitor in the treatment of HIV, is subject to active efflux transport mediated by ABCB1 (P-glycoprotein). Several coding variants of *ABCB1* have been described and associated with variable effects on substrate drugs pharmacokinetics. Here, we investigated the effect of the four most common coding *ABCB1* single nucleotide polymorphisms (i.e., c.1199G > A, c.1236C > T, c.2677G > T and c.3435C > T) on the intracellular accumulation of bictegravir. Using a previously validated HEK293 recombinant cell line model, we found decreased bictegravir intracellular concentrations in cell lines overexpressing ABCB1 as compared to control cell lines, in line with the known role of ABCB1 in bictegravir transport. However, we were unable to demonstrate any significant difference in bictegravir intracellular accumulation when comparing HEK293 cells overexpressing the wild type (1236C-2677G-3435C, 1199G) or the variant (1236C-2677G-3435T, 1236T-2677T-3435T or 1199A) proteins. These findings suggest that the *ABCB1* c.1199G > A and c.1236C > T-c.2677G > T-c.3435C > T variants have no or at least limited impact on the active transport of bictegravir by ABCB1.

## Introduction

Bictegravir is a second-generation integrase strand transfer inhibitor (INSTI) widely prescribed worldwide as an antiretroviral agent active against human immunodeficiency virus (HIV). The drug has been recommended as a first-line antiretroviral agent in combination with tenofovir alafenamide and emtricitabine, available as a single tablet regimen^1,2^.

The advent of second generation INSTIs such as bictegravir, dolutegravir and cabotegravir represents a significant advancement for the management of persons living with HIV, mainly due to their overall high efficacy and favorable tolerability profile, as reported in pivotal trials^3^. Those drugs share a common favorable pharmacokinetic profile, although interindividual variability (IIV) has been demonstrated^4–6^. Whether this IIV translates into significant variability in drug response (toxicity and/or efficacy) is subject of considerable interest. Indeed, since approval of second generation INSTIs, concerns have been raised regarding adverse events, including those reported with bictegravir. Abnormal weight gain is notably concerning among patients treated with dolutegravir or bictegravir^7,8^. While initially reported for dolutegravir^9^, neuropsychiatric adverse events are a leading cause of bictegravir discontinuation^10^, prompting consideration of a potential class effect^11^. Furthermore, in terms of virological response, despite the high success rate achieved with bictegravir-containing regimens, real-life studies have demonstrated that low-level viremia (LLV) defined by plasma viral load measured between 50 and 199 copies/ml, is not an infrequent event. Chen and colleagues described an incidence of LLV as high as 6.2 per 100 person-year of follow-up^12^. While a complete understanding of the mechanisms and causes of LLV is currently lacking, studies suggests that low drug tissue concentrations might be linked to residual viral transcription^13,14^, and limited intracellular concentrations in lymphoid tissue have been shown for INSTIs^15–17^.

Bictegravir is a known substrate of ABCB1 (also named P-glycoprotein, P-gp), an efflux transporter member of the ATP binding cassette and encoded by *ABCB1* gene^18,19^. Through its selective expression on the luminal side of enterocytes in the small intestine and colon, ABCB1 limits the absorption of several xenobiotics. It also exerts an excretory activity in the liver and kidneys through its expression at the canalicular pole of hepatocytes and the luminal membrane of renal tubular cells. Moreover, by being apically expressed in cells at the serosal side of blood-tissue barriers (e.g., blood–brain barrier or blood-placenta barrier), ABCB1 protects the so-called sanctuary organs from the accumulation of its substrates^20^. Eventually, the transporter plays an important role in the intracellular accumulation and pharmacokinetics of drugs. As such, ABCB1 deserves a particular attention in the field of HIV pharmacotherapy as it is expressed by peripheral blood mononuclear cells, including CD4 + T cells where the virus is known to replicate^21,22^.

Several single nucleotide polymorphisms (SNPs) have been described in *ABCB1*, but investigations into their functional impact have typically centered on a select few. Despite its low frequency, with minor allelic frequency estimated around 3% in European populations and generally lower in other ethnic groups^23^, the missense rs2229109 variant (c.1199G > A, p.Ser400Asn) has been subject of interest, as some studies have demonstrated a functional impact for the c.1199A variant^24–26^. The rs1128503 (c.1236C > T, p.Gly412Gly), rs2032582 (c.2677G > T, p.Ala893Ser), and rs1045642 (c.3435C > T, p.Ile1145Ile) variants are the three most frequent and unsurprisingly have been the most studied. These SNPs are in strong linkage disequilibrium, with the most frequent haplotypes in most ethnicities being the CGC (thereafter named wild-type, WT) and TTT combinations of variants at positions c.1236, c.2677 and c.3435, respectively^27^. Functional studies have focused on the effect of those individual SNPs or, more interestingly, of their haplotype combinations on drug transport, and have yielded variable results^28,29^.

Among antiretroviral agents, protease inhibitors have been the most studied drugs for the impact of *ABCB1* variants on drug transport^28^. A recent publication showed no effect of the four previously mentioned *ABCB1* variants on intracellular accumulation of darunavir^30^. The impact of *ABCB1* variants on bictegravir and other second generation INSTIs biodisposition remains currently unknown. Whether the most frequent *ABCB1* variants and their haplotypes significantly affect absorption, distribution and/or excretion of second generation INSTIs is however of particular interest as it could take part in the variability of their clinical response. The aim of this study is to investigate in vitro the impact of *ABCB1* c.1199G > A, c.1236C > T, c.2677G > T and c.3435C > T variants on the intracellular accumulation of bictegravir. Bictegravir was chosen for our in vitro study based on preliminary clinical pharmacokinetic assessments that indicated a higher intracellular accumulation of dolutegravir compared to bictegravir in PBMCs, suggesting that drug efflux transporters might play a more important and potentially more clinically impactful role in the transport of bictegravir.

## Results

### Flow cytometry

**Figure 1:**
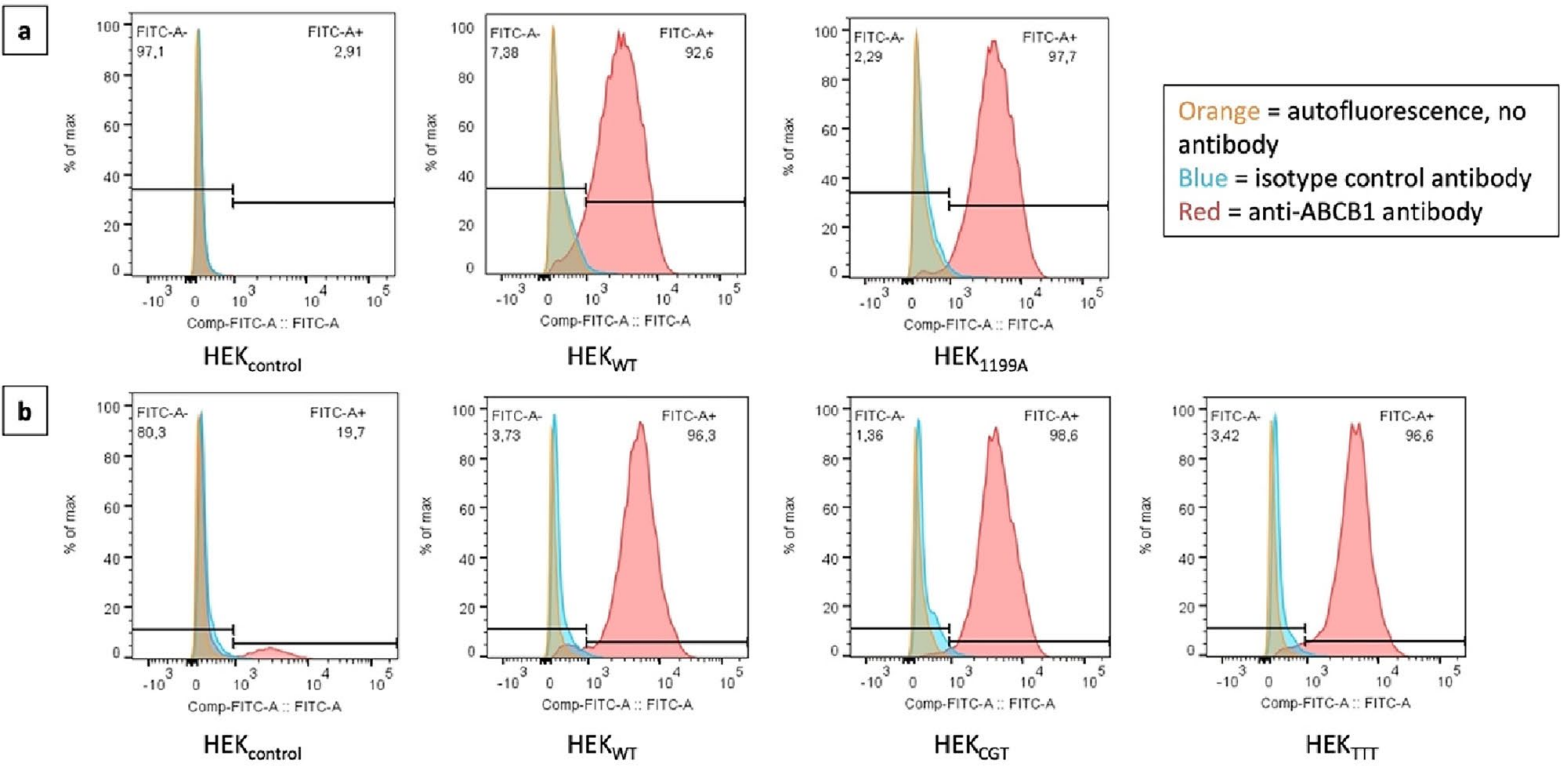
ABCB1 cell surface expression. Flow cytometry histograms of HEK293 cells transfected with (**a**) the empty pcDNA3.1 vector, *ABCB1*_*WT*_ or *ABCB1*_*1199A*_ and (**b**) the empty pcDNA3.1 vector, *ABCB1*_*WT*_, *ABCB1*_*CGT*_ or *ABCB1*_*TTT*_. Cells were incubated in the absence of antibody (autofluorescence, orange), with isotype control (blue) or with FITC anti-ABCB1 antibody (red).

Levels of ABCB1 expression in the different cell lines assessed in our models were compared using FACS with fluorescence parameters gated on the same level of intensity. Analytical flow cytometry confirmed similar levels of ABCB1 surface expression by the different recombinant cell lines (ranging from 92.6 to 98.6% of positivity). In the HEK_control_ batch used for c.1199G > A functionality assessment, no fluorescence was detected, confirming very low levels of ABCB1 basal expression. In the HEK_control_ batch, used as a control for experiments conducted with the haplotype, a small fluorescent peak was observed with ABCB1-antibody (19.7% positivity), indicating a low but detectable ABCB1 basal expression^31^ (Fig. 1).

### Functional assays

**Figure 2:**
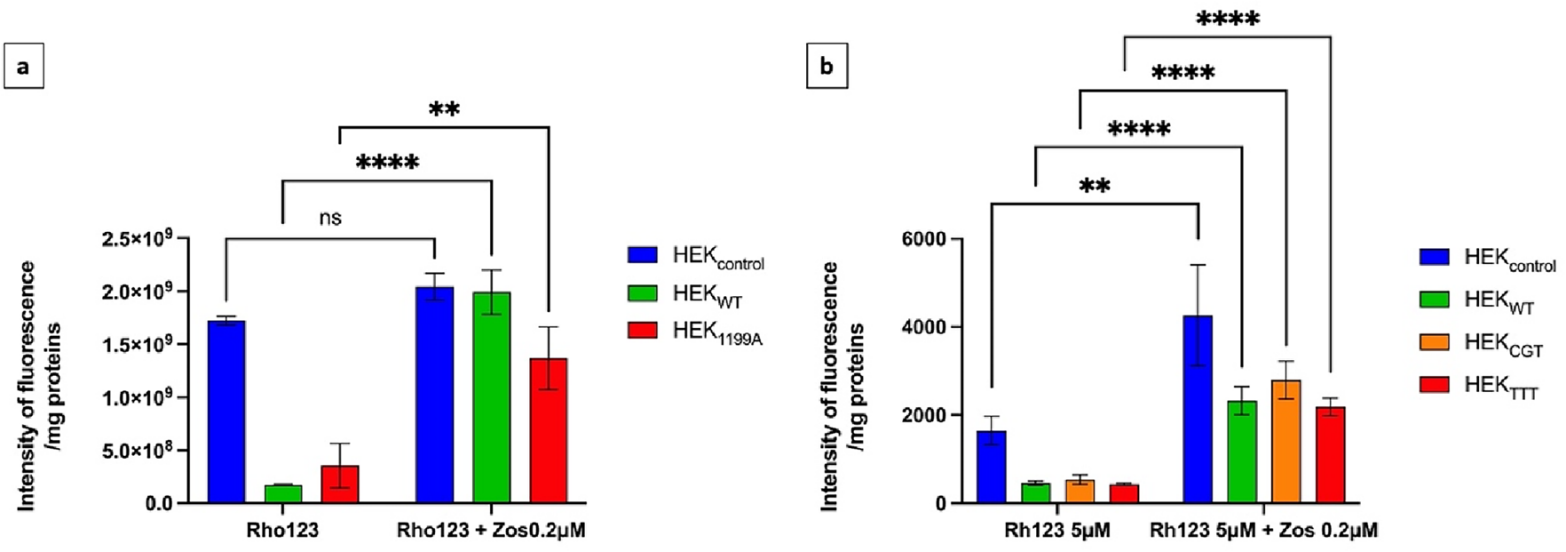
Model functionality assays using the ABCB1 substrate Rhodamine123 with or without the ABCB1 inhibitor Zosuquidar. Protein normalized intracellular fluorescence measured after incubation of (**a**) HEK_control_ and HEK_1199A_ and (**b**) HEK_control_, HEK_WT_, HEK_CGT_ and HEK_TTT_ with the fluorescent ABCB1 substrate Rhodamine123 (Rh123), in the absence (left) and in the presence (right) of Zosuquidar (Zos). Intensity of fluorescence is reported in arbitrary units. Results from a single experiment (N = 1 for (**a**) and N = 1 for (**b**)) reported as mean ± standard error (number of technical replicates performed per condition (n) = 3 for (**a**); n = 5 for (**b**)). Statistical analysis reported: ANOVA-2, after natural logarithmic transformation for (**b**). **p < 0.01; ****p < 0.0001.

Functionality of ABCB1 expressed by the transfected cell lines in our models was confirmed using Rh123, a known fluorescent ABCB1 substrate, and comparing its intracellular accumulation in the presence or the absence of zosuquidar, a potent and specific ABCB1 inhibitor. After cell incubation with Rh123, intracellular fluorescence was significantly lower in cell lines overexpressing ABCB1 (i.e., in HEK_WT_, HEK_1199A_, HEK_CGT_ and HEK_TTT_) compared to HEK_control_, expressing low or very low ABCB1 levels. When cell lines overexpressing ABCB1 had been pre-incubated with zosuquidar, intracellular fluorescence increased to a level close to the level measured for HEK_control_ (Fig. 2). A small but significant effect of the inhibitor was observed in the HEK_control_ batch used for haplotype functional characterization, likely reflecting the higher level of ABCB1 basal expression compared to the cellular batch used for c.1199G > A experiments.

### Impact of *ABCB1* c.1199G > A variant on the intracellular accumulation of bictegravir

**Figure 3:**
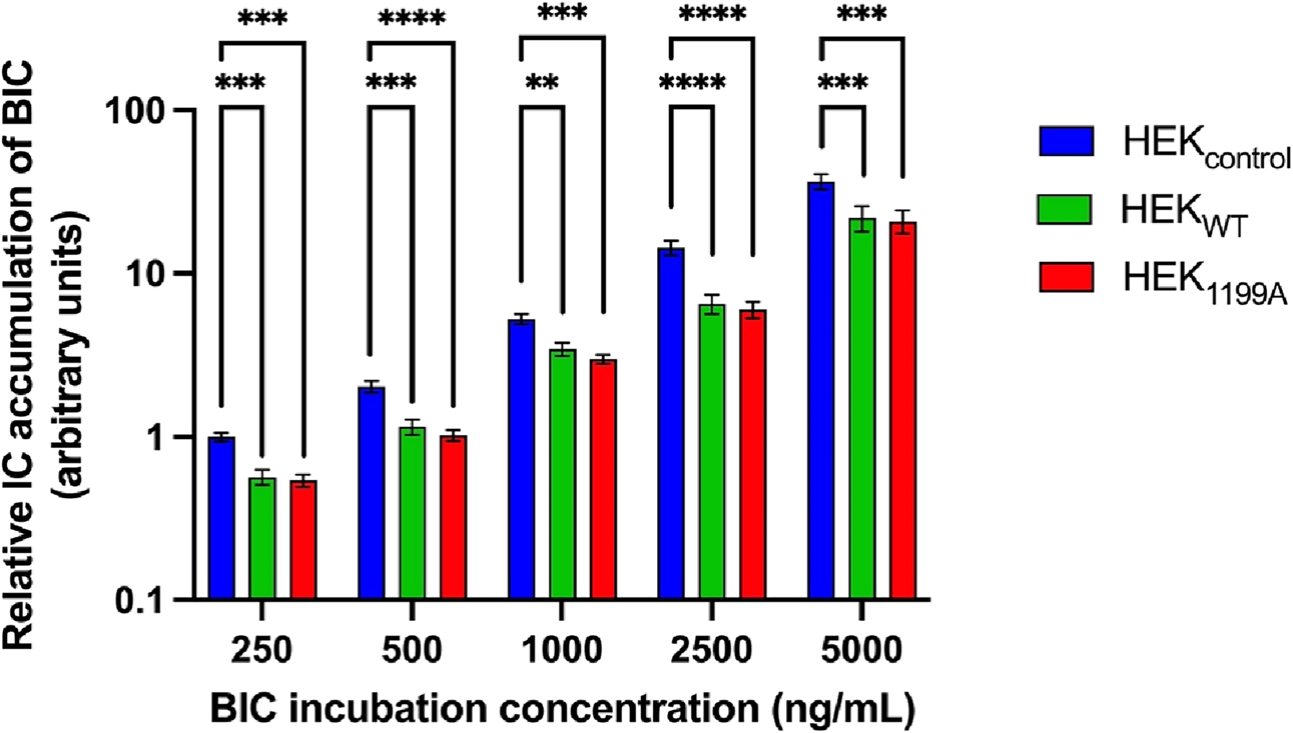
Intracellular protein-normalized bictegravir (BIC) concentrations in HEK_control_, HEK_WT_ and HEK_1199A_ cells after 2 h of incubation at several dose levels. Results of three experiments were pooled (N = 3). Protein normalized BIC intracellular concentrations are reported as fold-change of the mean of the protein normalized BIC intracellular concentration observed at the lowest BIC exposition concentration for HEK_control_ (number of technical replicates performed per condition and per experiment = 3)_._ Results reported as mean ± standard error. Statistical analysis reported: ANOVA-2 performed on pooled Ln-transformed data. **p < 0.01 ***p < 0.001 ****p < 0.0001.

The role of ABCB1 as a transporter for bictegravir and the impact of the *ABCB1* 1199G > A polymorphism on bictegravir transport was assessed by comparing the intracellular accumulation of bictegravir at different concentrations in the different cell lines (Fig. 3 and Supplementary Fig. 1). In accordance with what is expected for an ABCB1 substrate, bictegravir intracellular accumulations were significantly lower for HEK_WT_ and HEK_1199A_, both cell lines overexpressing ABCB1, than for HEK_control_. Our results did not show any significant difference of bictegravir intracellular accumulation between HEK_WT_ and HEK_1199A_. The transport of bictegravir by ABCB1 was confirmed in a separate experiment where HEK_1199G_ cells overexpressing the wild-type ABCB1, but not HEK_control_ cells, demonstrated a significant increase in intracellular accumulation of bictegravir in the presence of zosuquidar compared to without zosuquidar (Supplementary Fig. 2).

### Impact of *ABCB1* c.1236C > T-c.2677G > T-c.3435C > T variants on the intracellular accumulation of bictegravir

**Figure 4:**
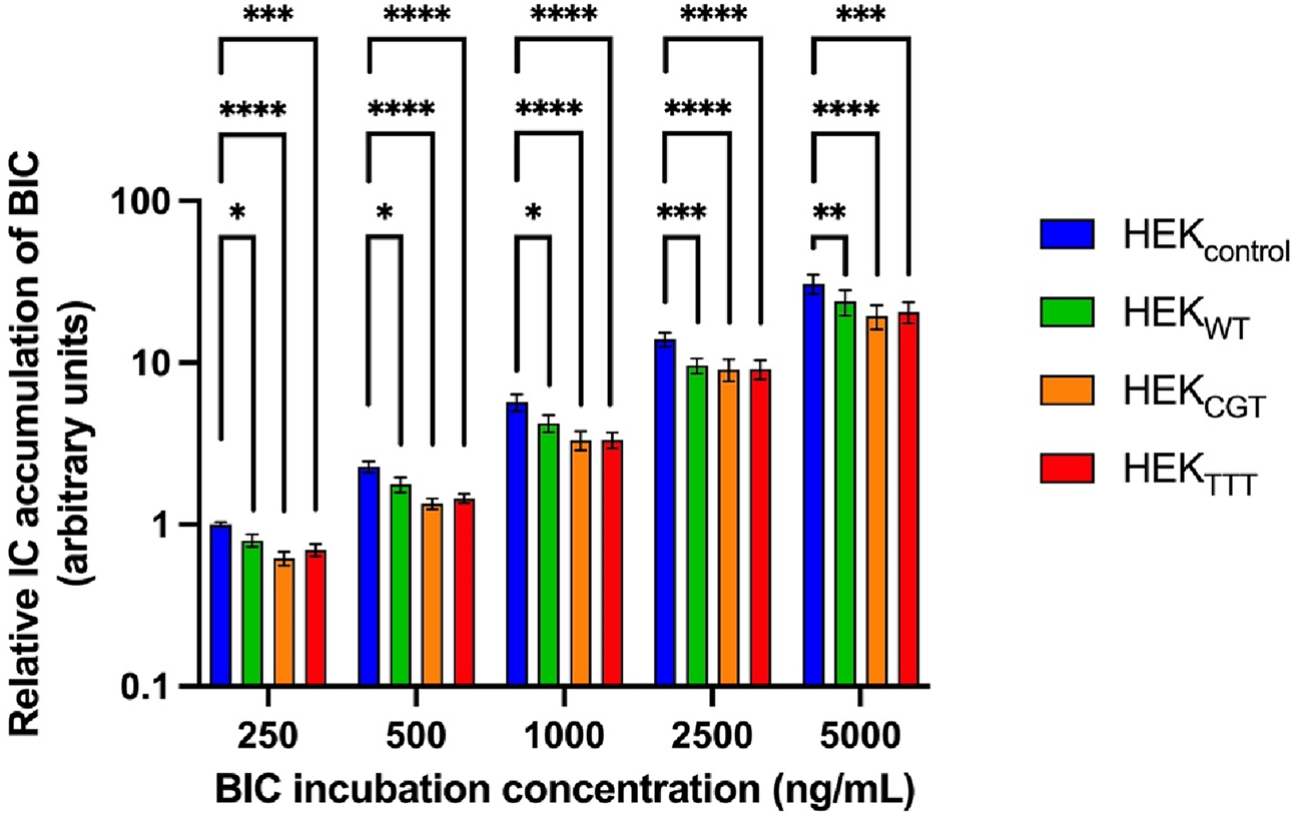
Intracellular protein-normalized bictegravir (BIC) concentrations in HEK_control_, HEK_WT_, HEK_CGT_ and HEK_TTT_ cells after 2 h of incubation at several dose levels. Results of three experiments were pooled (N = 3). Protein normalized BIC intracellular concentrations are reported as fold-change of the mean of the protein normalized BIC intracellular concentration observed at the lowest BIC exposition concentration for HEK_control_ (number of technical replicates performed per condition and per experiment = 3)_._ Results reported as mean ± standard error. Statistical analysis reported: ANOVA-2 performed on pooled Ln-transformed data. *p < 0.05, **p < 0.01, ***p < 0.001, ****p < 0.0001.

Next, the impact of the most frequent haplotypes linked to the *ABCB1* 1236C > T, *ABCB1* 2677G > T and *ABCB1* 3435C > T variants on bictegravir transport was assessed by comparing the intracellular accumulation of bictegravir in the different cell lines (Fig. 4 and Supplementary Fig. 3). Again, as expected for a substrate drug, intracellular accumulation of bictegravir was significantly lower for cell lines overexpressing ABCB1, namely HEK_WT_, HEK_CGT_ and HEK_TTT_, than for HEK_control_. Our results did not show any significant difference of bictegravir intracellular accumulation between the cell lines overexpressing the different *ABCB1* haplotypes (HEK_WT_ vs HEK_CGT_, HEK_WT_ vs HEK_TTT_ and HEK_CGT_ vs HEK_TTT_).

## Discussion

We report the findings of our in vitro experiments investigating the effect of four *ABCB1* variants on the intracellular accumulation of bictegravir, a second generation INSTI. Using a previously validated HEK293 recombinant cell line model, we found that bictegravir intracellular concentrations were significantly decreased in cell lines overexpressing ABCB1 as compared to control cell lines, in line with the known role of ABCB1 in the transport of bictegravir. However, we were unable to demonstrate any significant difference in intracellular accumulation between HEK_WT_ and HEK_1199A_ neither between HEK_WT_, HEK_CGT_ and HEK_TTT_. Those findings suggest that the *ABCB1* c.1199G > A and c.1236C > T-c.2677G > T-c.3435C > T variants have no or at least limited impact on the active transport of bictegravir by ABCB1.

Our results are consistent with an active transport of bictegravir by ABCB1. This finding was recently described using ABCB1-overexpressing Madin–Darby canine kidney strain II transfected cells compared with non-transfected cells^5^. Bictegravir is a lipophilic drug (LogD_7.4_ = 2.1) that belongs to the class II of the biopharmaceutical classification system (high permeability, low solubility). High transepithelial permeability has been shown for bictegravir and its high oral bioavailability is thought to be mainly due to passive diffusion^5^. Saturability of efflux transport has been considered likely, based on in vitro human colorectal adenocarcinoma (Caco-2) cell monolayers bidirectional permeability assays at high concentrations such as those associated to the therapeutic oral dose^5^. The extent to which in vivo intestinal absorption of bictegravir is affected by ABCB1 efflux activity remains unknown and caution should be exercised when using ABCB1 inducers or inhibitors.

On the contrary to the elevated drug diffusion from the intestinal compartment to the blood, low ratios of cerebrospinal fluid over plasma bictegravir concentrations have been demonstrated. This finding is probably mainly driven by the high plasma protein binding^32,33^. However, active efflux mechanisms may also play a role, and with low unbound plasma bictegravir concentrations, saturation of efflux transporters at blood-tissues barriers cannot be assumed. While lymphoid tissues have not been studied so far, limited bictegravir accumulation into human peripheral blood mononuclear cells has been recently shown^34^. The precise contribution of ABCB1 and other efflux transporters remains to be determined.

We investigated the impact of four important variants and their haplotype combinations, for which a body of evidence suggests a plausible effect on the drug transport by ABCB1, despite inconclusive effects reported by several in vitro and in vivo studies. Substrate-specific effects but also variations in experimental designs and unappreciated confounding factors are thought to explain at least partially this heterogeneity^20,28,29,35,36^.

The first variant investigated in this work is c.1199G > A (rs2229109), which leads to a serine to asparagine amino-acid substitution located close to the ABCB1 substrate binding site. Several reports have highlighted a substrate-specific effect of this SNP, possibly related to a differential effect on ABCB1 affinity towards its substrates^26,36^. An increase of ATPase activity was also shown for 1199A as compared to 1199G^24^.

The three other studied variants are in strong linkage disequilibrium, and we investigated the effect of the two most frequent haplotypes, being the wild-type CGC and the TTT combination. Studying haplotypes instead of isolated variants has been recommended as various SNPs can potentially have interacting effects^37^. The intermediate CGT haplotype was also included in the analysis, with the aim of deciphering the relative contribution of the c.3435C > T variant. Despite the latter (rs1045642) being a synonymous SNP, it has been shown to affect the structure and function of ABCB1. Different underlying mechanisms have been suggested, including changes in the folding and function of ABCB1 due to ribosome stalling, as translation necessitates the use of a rarer codon^38,39^. Other authors have suggested that this variant induces decreased mRNA stability and expression, probably related to an effect of the variant on mRNA secondary structure^40^. However, our recombinant cellular models have been generated to express a similar level of ABCB1, making them more suitable for testing the functional impact of the haplotype rather than its effect on mRNA expression and stability. On the contrary, the synonymous c.1236C > T (rs1128503) variant has not been shown to affect protein expression, neither protein function, although a putative effect on protein folding has been speculated^39^. The c.2677G > T variant (rs2032582) is non-synonymous and it has been suggested that the replacement of an alanine to a serine impacts the activity and substrate specificity of ABCB1, but not the protein expression^41^.

To the best of our knowledge, the effect of the studied variants on bictegravir transport has never been evaluated in vitro or in a clinical setting. In contrast, using the same cellular model and similar methods, our research group has previously demonstrated that the c.1199G > A variant has a significant impact on the intracellular accumulation of tacrolimus but not ciclosporin^26^. The same variant was also shown to affect the transport of imatinib, nilotinib and dasatinib^25^. Moreover, it was demonstrated that the c.1236C > T, c.2677G > T and c.3435C > T variants impact the anti-proliferative activity of tyrosine kinase inhibitors, with a more pronounced effect towards imatinib than nilotinib, dasatinib or ponatinib^42^. Lastly, the effect of the four variants were studied for darunavir, with no significant impact demonstrated^30^. Those different effects with different substrates but using similar in vitro methodologies point towards a substrate-dependent effect of the studied variants or their haplotype combinations, a hypothesis also supported by findings from other research groups using other methodologies for different substrates^43^, including HIV protease inhibitors, or anticancer drugs^44,45^. Importantly, as already stated, the clinical impact of those findings remains uncertain, as clinical studies have reported heterogeneous results^28,29^.

We believe that the absence of demonstrated significant effect of the most frequent *ABCB1* variants on the drug transport offers reassurance to the use of the drug in populations from different ethnic origins with different pharmacogenetic backgrounds. Although we cannot exclude that our assays were not powered to detect small differences between the different studied haplotypes, we believe those minor differences would probably not reach a level of clinical significance. One previously acknowledged limitation of our models, is that they assess the functional effect of the studied polymorphisms, and are not designed to evaluate variations in protein expression. As detailed previously, the polymorphisms that we studied have been associated with functional impacts (for c.1199G > A, c.2677G > T, c.3435C > T and possibly c.1236C > T) as well as possible expression impacts (for c.3435C > T) and as such our models are expected to be able to detect a significant impact of polymorphisms on drug transport by ABCB1. Further studies using other models assessing the effect of ABCB1 protein expression would usefully complement our approach.

In summary, we found that the second generation INSTI drug bictegravir accumulates significantly less in HEK293 transfected cell lines overexpressing ABCB1 than in transfected cell lines not overexpressing ABCB1, a finding consistent with the active efflux of bictegravir by ABCB1. We were unable to demonstrate any significant effect of the *ABCB1* c.1236C > T-c.2677G > T-c.3435C > T and c.1199G > A variants on bictegravir intracellular accumulation. Those findings are suggestive of an absence of functional impact, or at least a limited impact, of the aforementioned polymorphisms on bictegravir clinical pharmacokinetics. Future studies should evaluate the effect of frequent polymorphisms in other relevant bictegravir transporters such as ABCG2. Moreover, clinical studies should confirm our findings in vivo, and ideally assess the impact of the main polymorphisms of those different transporters, as well as the net effect of their combinations on bictegravir transport.

## Methods

All methods were carried out in accordance with relevant guidelines and regulations; in accordance with the Belgian legislation, no approval from the institutional ethical board was sought for this study.

### Chemicals and reagents

Dulbecco’s Modified Eagle Medium (DMEM), fetal bovine serum (FBS), penicillin/streptomycin, and enzyme-free cell dissociation buffer were purchased from Gibco (Thermo Fisher Scientific); G-418 from Roche; Poly-l-Lysine and Rhodamine (Rh123) from Sigma-Aldrich; flow cytometry antibodies (FITC mouse anti-human CD243, clone 17F9, reference 557002; and FITC mouse IgG2b κ isotype control, clone 27–35, reference 555742) from BD Biosciences; zosuquidar hydrochloride (LY335979) from Bio-connect; bictegravir from Alsachim (Schimadzu) and deuterated bictegravir (bictegravir-15N, d2) from Toronto Research Chemicals. All chemicals used in drug quantification were of analytical grade.

### Generation and characterization of recombinant cell lines

#### Generation of cell lines

The generation and characterization of recombinant cell lines have been described in previous work conducted by our lab^26,42^. Briefly, human embryonic kidney (HEK293) cells were purchased from American Type Culture Collection and transfected with plasmids carrying either wild-type *ABCB1* 1199G-1236C-2677G-3435C (thereafter named HEK_WT_) or several *ABCB1* variant combinations obtained using site-directed mutagenesis, 1199A-1236C-2677G-3435C (thereafter named HEK_1199A_), 1199G-1236C-2677G-3435T (thereafter named HEK_CGT_) and 1199G-1236T-2677T-3435T (thereafter named HEK_TTT_); or an empty vector (thereafter named HEK_control_). HEK_CGT_ was selected, in addition to HEK_WT_ (carrying the CGC haplotype) and HEK_TTT_, to test the potential impact of c.3435C > T variant. HEK293 cells feature low endogenous levels of expression of ABCB1, ensuring that ABCB1 expression originates almost exclusively from the transfection process^31^. This model has previously been extensively characterized using flow cytometry, Western blot, and fluorescence microscopy, and validated using reference substrates and inhibitors of ABCB1^26,42^. As the same cells were used, only flow cytometry and functional tests using reference substrates and inhibitors were used to re-characterize them.

#### Flow cytometry

After thawing, cells were put in culture for at least seven days in the presence of the selection antibiotic G418 (1 g/l). Cells were detached from culture plates, centrifugated and 0.5 × 10^6^ cells were counted, resuspended in 2 ml and washed twice with ice-cold fluorescence activated cell sorting (FACS) Buffer (PBS, FBS 1% (v/v), EDTA 1 mM). Cells were then re-suspended in FACS buffer supplemented with 10% anti-ABCB1 antibody or 10% isotype control or in buffer with no antibody, and left to incubate for 45 min on ice and in the dark. After a final wash followed by resuspension in FACS buffer, cells were characterized using a BD FACSVerse flow cytometer and sorted using a BD FACSAria III (BD Biosciences). Raw data were analyzed using FlowJo version 10.8.1 (Ashland).

#### Functional assays

0.05 × 10^6^ cells were seeded on poly-l-lysine coated 96-well plates and incubated overnight. The next day, Rh123 and zosuquidar dilutions were prepared from a stock solution. Cells were incubated for 90 min at 37 °C in the presence of Rh123 at 5 μM. The effect of ABCB1 inhibition was assessed by preincubation of the cells for 15 min with zosuquidar at a concentration of 0.2 μM. After incubation with Rh123, the supernatants were discarded. The cells were washed two times with ice-cold PBS. Sterile water was then added and detachment and lysis were performed through scratching of the well’s surface followed by sonication. Finally, fluorescence of Rh123 was analyzed by an iD3 Spectramax (Molecular Devices) fluorometer (c.1199G > A model validation) or a M3 Spectramax (Molecular Devices) fluorometer (haplotype model validation); excitation wavelength was set at 485 nm and emission at 530 nm. The solution was then used for protein quantification for results normalization.

### Cell culture

Transfected HEK293 cells were cultured at 37 °C and 5% CO_2_ in DMEM with high glucose, l-glutamine, and sodium pyruvate supplemented with 10% (v/v) FBS and 1% (v/v) penicillin–streptomycin. For re-culturing, the cells were detached with enzyme-free cell dissociation buffer and culture medium was changed at regular intervals.

### Intracellular bictegravir accumulation experiments

0.7 × 10^6^ cells were seeded on poly-l-lysine-coated 12-well plates and incubated overnight. The next day, bictegravir dilutions were prepared in DMEM from a stock solution and added in each well to obtain a final concentration of 0.25, 0.5, 1, 2.5 or 5 mg/l, and a final volume of 1 ml per well. These concentrations were chosen to cover the range of total plasma concentrations found in bictegravir-treated patients. Cells were incubated in triplicate at 37 °C in the presence of 5% CO_2_ for 120 min. Subsequently, plates were then centrifuged for 3 min at 4 °C, and maintained on ice throughout the experiment to block drug efflux. Cells were washed twice with 1 ml of ice-cold PBS. Supernatant was discarded and 400 μl of a mixture of methanol/water 60/40% (v/v) containing 20 ng/ml of bictegravir-15N, d2 as internal standard were added for drug extraction. The well’s surfaces were scratched followed by a 60 min agitation on a rocking platform at 4 °C. Cell suspensions were collected and set aside, while the wells were rinsed with 300 μl of sterile water. Cell suspensions were centrifugated for 10 min at 10,500×*g*, 4 °C and 100 μl of the supernatant was transferred to a vial for bictegravir liquid chromatography-tandem mass spectrometry (LC–MS/MS) quantification. The remaining fraction was combined with the 300 μl of water used to rinse the well. This mixed solution was used for protein quantification for results normalization.

### Drug quantification

Bictegravir intracellular concentrations were determined using an adapted version of a previously published LC–MS/MS method on a Xevo TQS-micro tandem quadrupole mass spectrometer (Waters)^46^. Calibrators ranging from 1 to 500 ng/ml of bictegravir concentrations, containing the internal standard solution at 20 ng/ml of dolutegravir-d4 and 20 ng/ml of bictegravir-15N, d2 were prepared. Chromatographic separation was achieved on a Waters UPLC BEH C18 1.7 μm column (2.1 × 50 mm) maintained at 40 °C. The injection volume was 10 μl and the flow rate was 0.5 ml/min. The mobile phase consisted of a gradient of water/formic acid 0.1% (mobile phase A) and acetonitrile (mobile phase B), starting with 95% A and 5% B, transitioning to 80% B over 6 min, returning to 5% B at 6.1 min and maintaining this ratio until the end of the run (total run time: 8 min). The following ion transitions were monitored: 450.1 > 289.1 for bictegravir and 453.2 > 289.2 for bictegravir-15N, d2.

### Protein quantification

Total proteins were quantified in pellets using the BCA kit (Thermo Fisher Scientific) according to the manufacturer’s instructions. Absolute bictegravir concentrations were normalized by total protein content.

### Statistical analysis

Statistical analyses were carried out using GraphPad Prism version 9.5.1 for Mac OS X (GraphPad Software). For the functional assays, protein normalized intracellular fluorescences were compared after log-transformation using the natural logarithm (Ln), when required. Comparison was performed using a two-way ANOVA (α = 0.05) followed by a post-hoc analysis testing the effect of the inhibitor, with Bonferroni correction for multiple testing. For the intracellular bictegravir accumulation experiments, triplicates were performed during each experiment, and each experiment was performed thrice. For each experiment, protein normalized bictegravir intracellular concentrations were reported as fold-change of the mean of the protein normalized bictegravir intracellular concentration observed at the lowest bictegravir exposition concentration for HEK_control_ during the same experiment. Fold-change results from the three biological replicates were pooled and are shown on Figs. 3 and 4. Statistical analysis were performed on Ln transformed data that were pooled using the same principle as described here above. Fold-changes were compared using a two-way ANOVA (α = 0.05) followed by a post-hoc analysis comparing fold-changes at each dose level, with Bonferroni correction for multiple testing.

### Supplementary Information

Supplementary Information.

## Data Availability

The data collected from this study are available from the corresponding author on reasonable request.
