# Supplementary material for: Effect of ABCB1 most frequent polymorphisms on the accumulation of bictegravir in recombinant HEK293 cell lines
Source: Sci Rep. 2024 Jul 15;14:16290. doi: 10.1038/s41598-024-66809-0 (PMC11251168; doi:10.1038/s41598-024-66809-0)
Supplement: Supplementary file 1 — Supplementary Information. [file 41598_2024_66809_MOESM1_ESM.pdf]

Effect of ABCB1 most frequent polymorphisms on the accumulation of bictegravir in recombinant HEK293 cell lines

Julien De Greef, Mathilde Akue, Nadtha Panin, Kévin-Alexandre Delongie, Marina André, Gwenaëlle Mahieu, Emilia Hoste, Laure Elens, Leïla Belkhir*, Vincent Haufroid*

Supplementary information

***Supplementary Figure 1*: Impact of ABCB1 c.1199G>A variant on the intracellular accumulation of bictegravir**

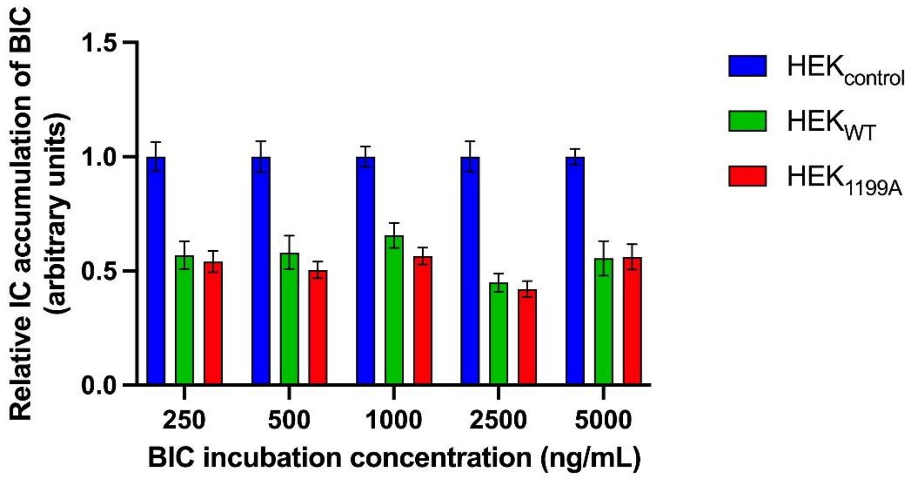

Results of three experiments were pooled (N=3). Protein normalized BIC intracellular concentrations are reported as fold-change of the mean of the protein normalized BIC intracellular concentration observed at each concentration for $HEK_{control}$ (number of technical replicates performed per condition and per experiment = 3).

Supplementary Figure 2:

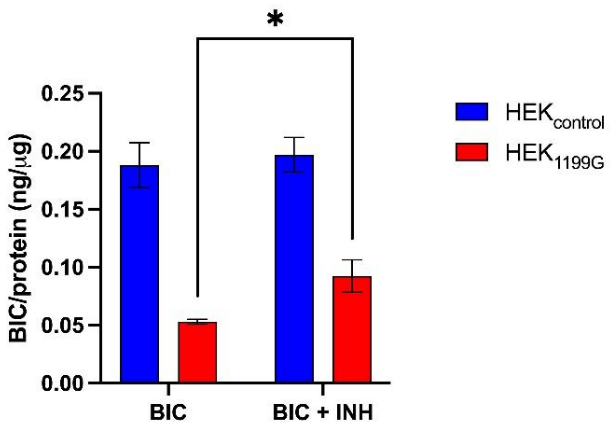

Effect of Zosuquidar 0,2µM (INH) on bictegravir (BIC) intracellular accumulation

HEK293 cells were exposed to BIC at 2500ng/mL

Methods

0.7x10[6] cells were seeded on poly-l-lysine-coated 12-well plates and incubated overnight. The next day, bictegravir and zosuquidar dilutions were prepared from a stock solution. Cells were incubated for 120 minutes at 37 °C (5% CO_2) in the presence of bictegravir at 2500 ng/mL. The effect of ABCB1 inhibition was assessed by preincubation of the cells for 15 minutes with zosuquidar at a concentration of 0.2 µM. Subsequently, plates were then centrifuged for 3 min at 4 °C, and maintained on ice throughout the experiment to block drug efflux. Cells were washed twice with 1 mL of ice-cold PBS.

Drug extraction was then performed as described in the Manuscript, in the Methods section describing Intracellular bictegravir accumulation experiments. Drug quantification and protein quantification were performed as described in the dedicated Methods sections.

Reported statistical analysis: ANOVA-2 with post-hoc Bonferroni correction test, performed on log-transformed data.

Supplementary Figure 3: **Impact of ABCB1 c.1236C>T-c.2677G>T-c.3435C>T variants on the intracellular accumulation of bictegravir.**

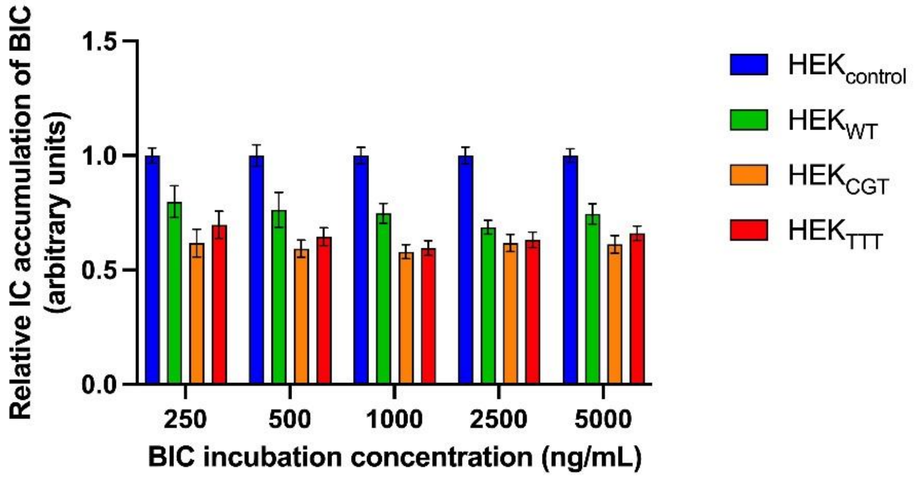

Results of three experiments were pooled (N=3). Protein normalized BIC intracellular concentrations are reported as fold-change of the mean of the protein normalized BIC intracellular concentration observed at each concentration for HEK$_{control}$ (number of technical replicates performed per condition and per experiment = 3).
